# Analytical Model of Large Data Transactions in CoAP Networks

**DOI:** 10.3390/s140815610

**Published:** 2014-08-22

**Authors:** Alessandro Ludovici, Piergiuseppe Di Marco, Anna Calveras, Karl H. Johansson

**Affiliations:** 1 Wireless Network Group (WNG), Department of Telematics Engineering, Universitat Politècnica de Catalunya, C/Jordi Girona 1-3, Mòdul C3, 08034 Barcelona, Spain; E-Mail: alessandro.ludovici@antel.upc.edu; 2 i2CAT Foundation, Gran Capità 2-4 (Nexus Building), 08034 Barcelona, Spain; 3 School of Electrical Engineering, Royal Institute of Technology, 100 44 Stockholm, Sweden; E-Mails: pidm@kth.se (P.D.M.); kallej@kth.se (K.H.G.)

**Keywords:** CoAP, 6LoWPAN, analytical model, fragmentation, Internet of Things, IEEE 802.15.4

## Abstract

We propose a novel analytical model to study fragmentation methods in wireless sensor networks adopting the Constrained Application Protocol (CoAP) and the IEEE 802.15.4 standard for medium access control (MAC). The blockwise transfer technique proposed in CoAP and the 6LoWPAN fragmentation are included in the analysis. The two techniques are compared in terms of reliability and delay, depending on the traffic, the number of nodes and the parameters of the IEEE 802.15.4 MAC. The results are validated trough Monte Carlo simulations. To the best of our knowledge this is the first study that evaluates and compares analytically the performance of CoAP blockwise transfer and 6LoWPAN fragmentation. A major contribution is the possibility to understand the behavior of both techniques with different network conditions. Our results show that 6LoWPAN fragmentation is preferable for delay-constrained applications. For highly congested networks, the blockwise transfer slightly outperforms 6LoWPAN fragmentation in terms of reliability.

## 1. Introduction

In the recent years, the Internet of Things (IoT) has emerged as one of the most promising developments of the Internet of the future. According to the IoT vision [1], devices with Internet connectivity will be embedded in the physical environment and in everyday objects. Wireless Sensor Networks (WSNs) constitute a key element of the IoT. The small size, low cost and low energy consumption of sensor devices, in fact, allows WSNs to be easily deployed in a variety of environments.

Monitoring and sensing of common physical variables as temperature, pressure or humidity are typical application of WSNs. They are characterized by having slow changes of state and, therefore, require a sensor node to sample their value at low rates. Generally, the information collected by each node is constituted by few bytes and fits the data frames defined by most common WSNs standard protocols such as the IEEE 802.15.4 protocol [2], which is the *de facto* standard for MAC and physical layers of WSNs. A Maximum Transferable Unit (MTU) of 127 bytes is allowed for IEEE 802.15.4 data frames. The presence of the header as well as of security mechanisms reduces the payload to 87 bytes. Further overhead is created by upper layer protocols, which can reduce significantly the space available for application data.

Monitoring applications of physical variables that have frequent changes of state could not be compatible with the constraint of the IEEE 802.15.4 MTU. Typically sensor nodes sample and store the information in a data log that is later sent to a sink node. The size of the data log, however, does not fit with the packet constraints of WSN protocols. Example of such applications can be found in [3–5]. In [3] a WSN has been deployed to monitor railway vibrations and produces packets of 7 KB per node. In [4] the authors present a WSN application for structure health monitoring where each node produces data logs of 512 KB. A WSN used to monitor a volcano activity [5] produces data logs of 256 bytes per node. IoT protocols, therefore, should be able to deal with large packets.

The IETF has standardized the use of the IPv6 protocol in IEEE 802.15.4 networks. The resulting standard is known as IPv6 over Low Power Personal Area Networks (6LoWPAN) [6]. 6LoWPAN introduces an adaptation layer that allows IPv6 datagrams to meet the requirements of the IEEE 802.15.4. This layer provides a fragmentation mechanism for the packets that exceed the MTU of the IEEE 802.15.4 standard. 6LoWPAN fragmentation allows splitting a packet into many fragments, which are sent individually in 802.15.4 data frames. The reception of each frame relies on the link layer acknowledgment (MAC ACK) as defined in [2]. The link layer, however, is not able to distinguish if a data frame is part of a fragmented packet or not. Consequently, if a fragment is lost, then the subsequent fragments are sent, although it is not possible to reconstruct the packet. As a consequence, the whole fragmented packet has to be retransmitted. The Constrained Application Protocol (CoAP) [7] instead allows overcoming this drawback by enabling reliable transmission at application layer.

CoAP is a protocol defined by the IETF for WSN applications such as smart energy and building automation. CoAP implements the key features of HTTP while adding its own mechanisms to best adapt to WSN characteristics. Among them, CoAP defines an alternative to 6LoWPAN fragmentation, which is called CoAP blockwise transfer [8] to enhance reliability in large data transactions and avoid 6LoWPAN fragmentation. A packet is divided into blocks and the data transfer into multiple request/response transactions. In this sense, the transmission of a single block corresponds to a single CoAP request/response transaction. This provides reliable data transactions using CoAP confirmable (CON) messages. A node receiving a CON message must acknowledge its reception to the source node. Should the source node not receive a CoAP acknowledgment (CoAP ACK), it retransmits the request. Then, the failure of a single block causes only the retransmission of the relative request. Instead, a fragmented 6LoWPAN packet is sent in a unique CoAP request/response transaction. The CoAP ACK, therefore, is sent after that all the fragments have been received.

Although the blockwise transfer is designed to improve the reliability and reduce the number of retransmitted packets for a dedicated client-server transmission, its effect on reliability and delay in a contention-based network scenario may not be always positive. In fact, due to the transmission of a CoAP ACK for each block, the CoAP blockwise transfer causes a higher traffic in the network and therefore a higher level of contention at MAC layer.

In the literature there are only few works that focus on CoAP blockwise transfer and 6LoWPAN fragmentation. The study in [9] evaluates the effects of 6LoWPAN fragmentation on the energy consumption. An analytical model of 6LoWPAN route-over forwarding mechanism is considered in [10], which includes the presence of 6LoWPAN fragmentation. CoAP blockwise transfer is considered in [11,12]. In [11], an experimental assessment of the performance of CoAP with blockwise transfer is illustrated. As a preliminary result, it is shown that the medium access contention has a strong impact on the performance. In [12], the authors present a service management system that seeks to reduce the energy consumed by blockwise transfer. However, to the best of our knowledge, there is no analytical study available that assesses the performance and the fundamental limits of 6LoWPAN fragmentation and CoAP blockwise transfer when used in contention-based scenarios. A thorough study of both techniques is therefore needed to understand their behavior and optimize their use in WSNs.

In this paper, we propose a novel analytical model to study the CoAP blockwise transfer and 6LoWPAN fragmentation in WSNs. The main contribution is the possibility to understand and compare the behavior of both techniques with different network conditions.

We consider reliability and delay as performance indicators. Both techniques are evaluated using the CoAP observe model [13]. This allows a client node to register to a resource exposed by a server node and to receive updates of its states. The application of this model helps to reduce the request/response interaction and it is particularly useful in WSN monitoring applications.

Our model considers the presence of the Carrier-Sense Multiple Access with Collision Avoidance (CSMA/CA) mechanism defined by the IEEE 802.15.4 standard. The inclusion of this mechanism is instrumental to understand the interaction between higher-layer protocols such as 6LoWPAN and CoAP with the IEEE 802.15.4. In particular, it allows evaluating the packet losses caused by collisions and channel access failures. The study in [14] is used as a basis for the analysis of the CSMA/CA mechanism. Its application to our study presents several challenges. Although it is typically assumed that the application layer generates packets following a known traffic distribution (e.g., the Poisson distribution), the subsequent fragmentation or block division implies a bursty transmission at MAC layer. We refer to this traffic condition as ‘mixed’ traffic. At MAC layer, in fact, only the arrival of the first fragment or block follows a Poisson distribution. The arrival of the remaining fragments or blocks is characterized by a saturated traffic condition. Therefore, moving forward from the assumption made in [14], the busy channel probability and the collision probability depend on the transmission stage. The analysis of this behavior requires adapting the original CSMA/CA model to the new traffic conditions. Existing works on the IEEE 802.15.4 MAC protocol have considered both saturated traffic (*i.e.*, when node queues are always non-empty) and unsaturated traffic conditions (e.g., [14–16]). Bursty ON-OFF traffic conditions have only been considered in [17]. None of the existing works focus on the presence of mixed traffic conditions in the analysis of the IEEE 802.15.4 MAC protocol, which is a further contribution of this paper.

In the next section we review 6LoWPAN fragmentation, CoAP blockwise transfer and the unslotted CSMA/CA mechanism as defined by the IEEE 802.15.4 standard. The rest of the paper is organized as follows: in Section 3 we review and present the adaptation of the analytical model presented in [14]. The proposed model of 6LoWPAN fragmentation and blockwise transfer is illustrated in Section 4. In Section 5 we validate the model by Monte Carlo simulations and present the results of the performance evaluation. Finally, in Section 6 we conclude the paper and give some guidelines for future works.

## 2. Background

In this section, we present 6LoWPAN fragmentation and CoAP blockwise transfer. Both techniques can be used in CoAP data transactions. As mentioned, they allow transmitting packets that do not fit in a single IEEE 802.15.4 frame. Finally, we review the unslotted CSMA/CA mechanism as defined by the IEEE 802.15.4 standard.

### 2.1. 6LoWPAN Fragmentation

**Figure 1.**
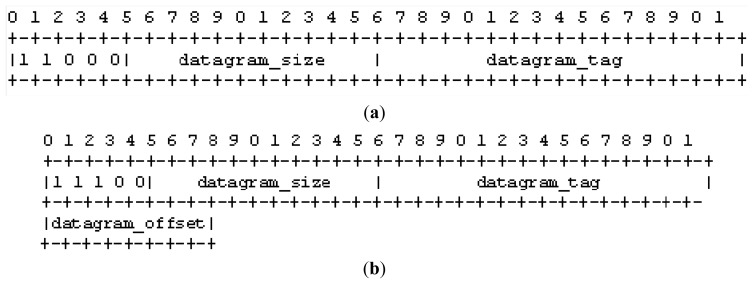
6LoWPAN Fragment headers. (**a**) First Fragment; (**b**) Second Fragment.

6LoWPAN introduces an adaptation layer between network and data link layers. Its primary function is to encode the IPv6 header and to fragment the packets that exceed the MTU of the MAC layer. Should a packet need to be fragmented, 6LoWPAN appends a fragmentation header [6] to each fragment of the original packet. Two distinct headers are used to indicate whether the fragment is the first or it is one of the subsequent. The main difference relies in the presence of a header field that indicates the offset of the fragmentation. This is present only in subsequent fragments. Figure 1 shows the fragmentation header for the first fragment and the subsequent fragments.

The destination node of the fragments uses the source and destination addresses of the 802.15.4 header plus the *datagram_size* and *datagram_tag* to identify the fragments that belong to a given 6LoWPAN packet. The fragment reassembling process will produce the original packet with the size specified in the *datagram_size* field. In the reassembling process, the node uses the *datagram_offset* field to determine the location of the fragment within the original packet.

**Figure 2.**
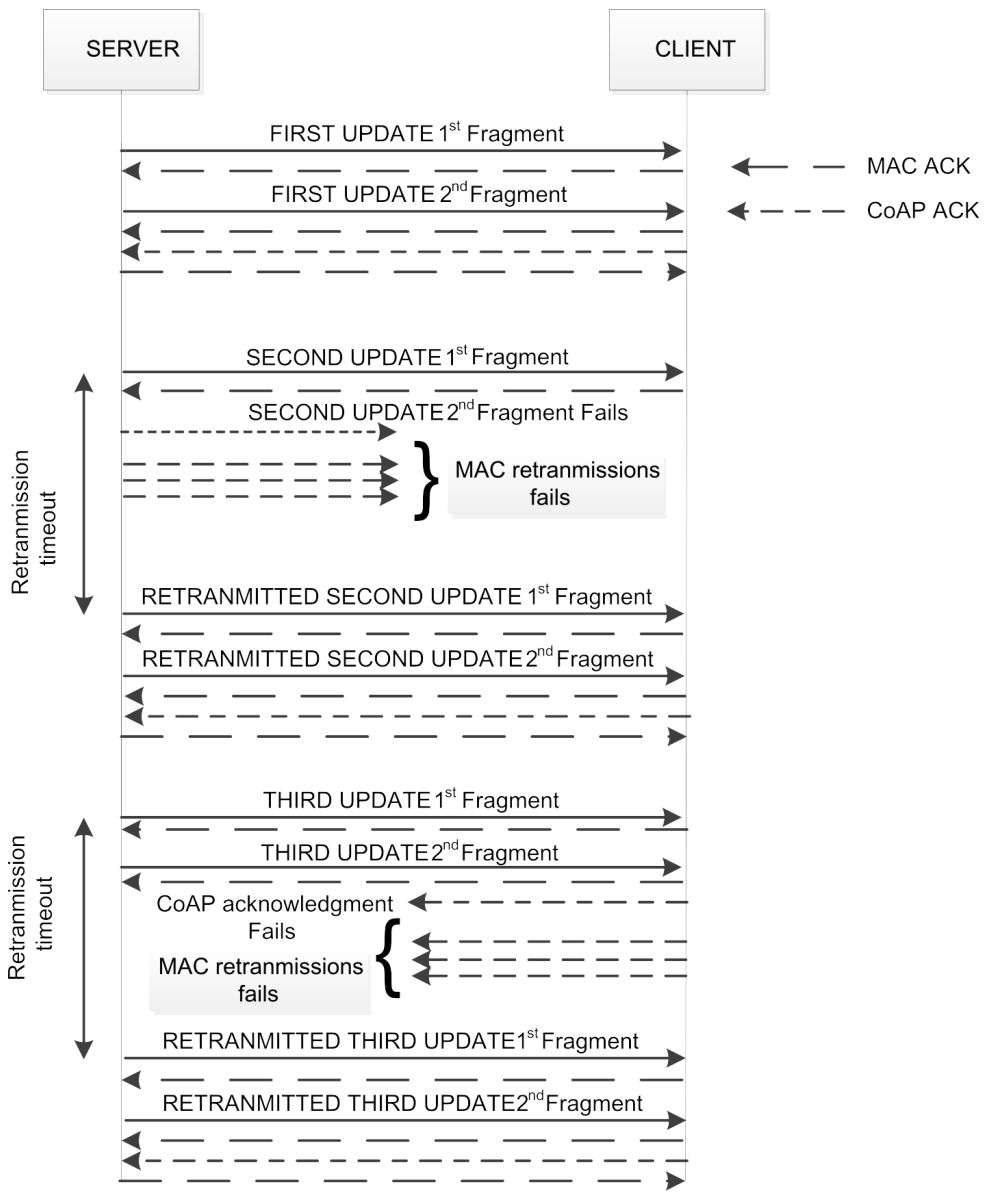
Each update is composed by two fragments. The client fails to send the CoAP ACK at the third update while a fragment transmission fails in the second one. Both failures cause the retransmission of the entire fragmented update.

As mentioned, in this paper we consider CoAP data transactions that uses the observe model. Figure 2 shows the transfer of an observe update using 6LoWPAN fragmentation. The server sends the update using CoAP CON messages. Each update is composed by *F* = *2* fragments. The destination node acknowledges the correct reception of a fragment with a MAC ACK. Moreover, it sends a CoAP ACK to acknowledge the reception of the whole fragmented update. This is sent only after the client receives correctly the last fragment. Should the destination node not receive one of the *F* fragments or the CoAP ACK transmission fails, the source node retransmits all the *F* fragments up to four times, as specified in [7]. In the rest of this paper, we indicate with *c* the maximum number of retransmissions allowed in CoAP.

With reference to Figure 2, the server retransmits the second update after the expiration of the CoAP retransmission timeout (RTO) [7], which is consequent to the failure of both the transmission of the second fragment and that of the relative MAC retransmissions. The update is then retransmitted successfully. The third update instead, is retransmitted after all the MAC retransmissions of the CoAP ACK fail.

### 2.2. CoAP Blockwise Transfer

Blockwise transfer enables the transmission of large CoAP packets in separated blocks. It is activated including the block option [8] in the CoAP header. CoAP defines two block options: *block_1* and *block_2*. Their use depends whether the payload is present in a response to a GET request (block_2) or in the POST or PUT request (block_1).

**Figure 3.**
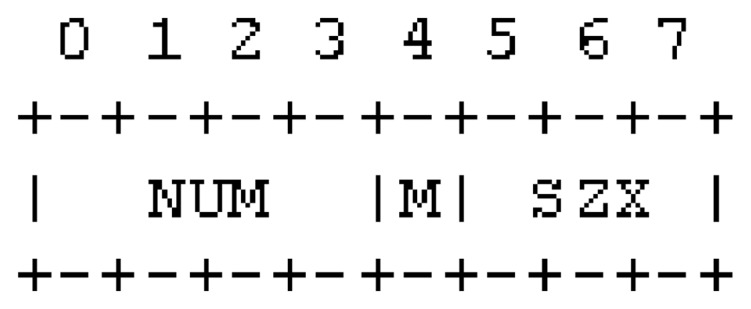
Encoding of the block option.

As illustrated in Figure 3, the block option contains three kind of information: the size of the block (SZX), a flag to indicate if more blocks are following (M) and the sequence number of the block (NUM). The SZX and M fields have fixed size. The NUM field can have three different sizes: 4, 12 or 24 bits.

The observe update is divided in *B* blocks that are transferred in multiples request/response transactions. The transmission of a block is consequent to the reception of the relative request. Each request must contain the *block_1* option. In this case, the NUM field indicates the number of the block that the client is expecting to receive. The M field is equal to zero while the SZX field is used to indicate the desired size of the block. The size of the blocks, in fact, can be negotiated between the client and server. In the proposed model, we consider a block size that allows filling an entire IEEE 802.15.4 frame. This size corresponds to that of a single 6LoWPAN fragment. A greater size implies the use of 6LoWPAN fragmentation to send a single block, which would not allow analysing the behavior of CoAP blockwise transfer. On the other hand, a smaller block size does not allow comparing equally 6LoWPAN fragmentation and CoAP blockwise transfer. Please note that 6LoWPAN fragmentation could be considered as a generalization of blockwise transfer with a block size equal to the size of the un-fragmented packet.

Blockwise transfer can be used in combination with the observe option. In this case, the first block of an observe update is sent without the initial request of the client. Then, the server sends the subsequent block after the client acknowledges the previous one. Both the initial observe registration request and the relative response can contain the block option. The client, in fact, can request the use of blockwise transfer including the block option in the observe request. The server could acknowledge an observe request including the block option to indicate that it could send updates using the blockwise transfer.

**Figure 4.**
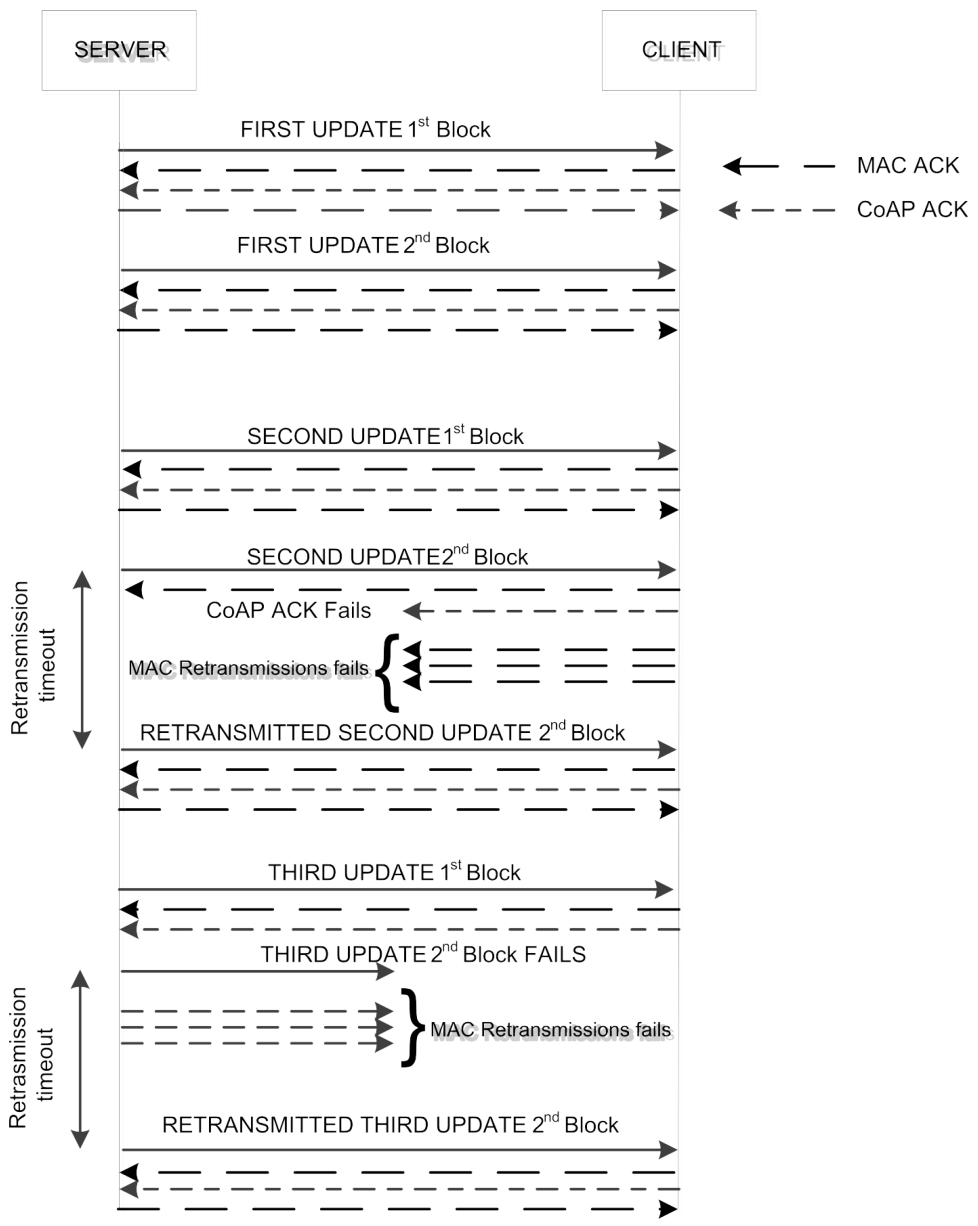
Blockwise transfer in observe data transaction. Two blocks compose each update. In the second update the CoAP ACK sent by the client fails while in the third one the second block fails. Both failures cause the retransmission of the relative block.

Figure 4 shows the use of the blockwise transfer in an observe transaction composed by three updates. The transmission of the CoAP ACK relative to the second block of the second update fails as well as the MAC retransmission. Therefore, the server retransmits the CoAP ACK after the expiration of the RTO. In the transmission of the third update, the second block transmission and the relative MAC retransmissions fail. As a consequence, the block is retransmitted after the expiration of the RTO.

### 2.3. Overview of the Unslotted CSMA/CA

The unslotted CSMA/CA mechanism is located at MAC layer. Its process is regulated by three variables, which are the number of backoff *NB*, the backoff exponent *BE*, and the retransmissions counter *RT*. When a node wishes to transmit a packet, *BE* is settled to its minimum value (*macMinBE)* while *NB* and *RT* are initialized to zero. The channel access is divided in two steps: a backoff period and the Clear Channel Assessment (CCA). In the backoff period, the MAC layer delays for a random number of *aUnitBackoffPeriod* units in the range [0, *W_k_*] = [0, 2*^BE^* − 1] where *W_k_* is the boundary of the backoff window and *k* the index representing the backoff stage. At the end of this period, the node performs the CCA. This is used to sense if the channel is busy or idle. During CCA, the node is in listening mode. It takes *aTurnaroundTime* units to switch to transmitting mode. Should the CCA fails, the values of NB and BE are incremented by one and up to a maximum value of *macMaxCSMABackoffs* and *macMaxBE* respectively. Should *BE* reach *macMaxBE*, it remains at this value until it is resettled. If *NB* exceeds *macMaxCSMABackoffs,* the transmission is aborted and packet is discarded due to channel access failure. In the other cases, the CSMA/CA algorithm generates a random number of backoff periods and repeats the process. If the channel is sensed to be clear, the node starts the transmission of the packet. After it is completed, the node waits for the MAC ACK. The reception of the MAC ACK is interpreted as a successful transmission. Should the node not receive the MAC ACK due to collision or MAC ACK timeout, the variable *RT* is increased by one up to *macMaxFrameRetries*. If *RT* is less than this values, the MAC layer initializes *BE* to its default value of macMinBE and repeats the CSMA/CA mechanism. The packet is discarded due to the retry limit when *RT* reaches its maximum value. In the rest of the paper, we denote by *m_0_* = *macMinBE*, *m_B_* = *macMaxBE*, *m* = *macMaxCSMABackoffs* and *n* = *macMaxFrameRetries*. A list of the main symbols used in this paper is reported in Table 1.

## 3. Traffic Generation Model and CSMA/CA Model

In this section we present the adaptation of the analytical model presented in [14]. The IEEE 802.15.4 standard defines the use of unslotted CSMA/CA in non beacon-enabled networks and slotted CSMA/CA in beacon-enabled networks. In this paper, we focus on non beacon-enabled networks using unslotted CSMA/CA. This MAC modality is of major interest in the standardization of IETF protocols [6]. We evaluate the protocol equations for a star topology, since this is the reference scenario recommended for CoAP application such as building automation and smart energy. A contention-based multiple access star topology allows for data collection from sensors to a gateway (network coordinator), that is reached with one-hop communication. Data communication through a multi-hop topology is envisioned in CoAP, but this is typically handled by using scheduled-based multiple access protocols. The extension of the model to this general scenario is interesting but not trivial and it is left as a future work. In particular, the performance of both 6LoWPAN fragmentation and CoAP blockwise transfer depend on the forwarding technique through relay nodes [18].

### 3.1. Traffic Generation Model

A major contribution of our model, with respect to [14] and the related literature, is the analysis of the performance of the MAC layer under bursty traffic conditions introduced along with 6LoWPAN fragmentation and CoAP blockwise transfer, as we detail as follows.

We assume that the CoAP layer generates observe updates with a Poisson distribution with rate λ. This is typical assumption in the related literature (e.g., [14–17]) to model traffic generation in WSN scenarios. As mentioned, each update is divided into *F* fragments or *B* blocks. Each fragment or block is included into a MAC frame of length *L* while the CoAP ACK has length *L_ACK_*. Both frames are transmitted using the unslotted CSMA/CA mechanism of the IEEE 802.15.4 standard. Moreover, we consider low traffic generation, *i.e.*, the traffic generation period for each node is long compared to the time it takes to forward an update, λ_1_ ≪ 1/(L × F), which is consistent with the minimum RTO of 1 s recommended by CoAP [7]. With higher rates the retransmission mechanism of CoAP could not be used. The RTO, therefore, represents a lower bound to the traffic generation period.

At MAC layer, the traffic arrival is characterized by a Poisson distribution of parameter λ*_l_* for the first fragment and by bursty traffic for the following *F-1* fragments or *B-1* blocks. The probability of generating the first fragment or block of an update at node *l* in a unit time *S_b_* is derived as:

(1) $$q_{l}=1-e^{\left(\lambda_{l}/S_{b}\right)}$$

In the rest of the paper we consider *S_b_* = *aUnitBackoffPeriod* as the basic unit time as in [14]. We recall that it corresponds to the transmission time of 20 symbols [2].

The probability of generating a new fragment or block after the previous one has been acknowledged or discarded is 1, until the last fragment or block has been acknowledged.

### 3.2. Analytical Model of the CSMA/CA Mechanism

In this section, we develop a generalized model of the IEEE 802.15.4 MAC considering the presence of 6LoWPAN fragmentation and CoAP blockwise transfer. The analysis aims at deriving the reliability as the probability of successful frame reception and the delay for successfully received frames. Both are relative to the MAC layer and will be included in performance indicator expressions for the CoAP layer, which are presented in the next section.

The analysis is based on the Markov chain model presented in [14] that accounts for the presence of heterogeneous traffic with different node packet generation rates and hidden terminals.

We first determine the CCA probability *τ_l_*, namely the probability that node *l* performs the carrier sensing procedure in a randomly chosen time unit. For each generated fragment, the CCA probability accounts for the number of times the CCA procedure is repeated due to busy channel and retransmissions, *i.e.*,

(2) $$\tau_{l}=\sum_{i=0}^{m}\prod_{j=0}^{i}\left(\alpha_{l,j}\right){\sum_{k=0}^{n}\left(\left(1-\sum_{j=0}^{m}\alpha_{l,j}\right)P_{\text{coll},l}\right)}^{k}b_{0,0,0}^{\left(l\right)}$$

where α*_l,j_* is the busy channel probability of node *l* during the *j-th* backoff stage, ${\text{b}}_{0,0,0}^{\left(\text{l}\right)}$ is the MAC frame generation probability, and P_coll,l_ is the collision probability, which we derive next.

The first sum determines the expected number of accesses for each transmission, the second sum accounts for the expected number of re-transmissions for each packet.

For unsaturated traffic conditions, the frame generation probability at MAC layer is:

(3) $${b_{0,0,0}}^{\left(l\right)}=\;\left(q_{l}\times F\right)$$

When traffic gets saturated, the MAC frame generation probability is calculated by the normalization condition of the corresponding Markov chain, as detailed in Proposition 4.1 in [14].

The busy channel probability due to packet transmission for the first fragment or block is the probability that no other node accessed the channel and found it idle in the previous *L* time units. After the previous fragment or block has been acknowledged at MAC layer, the node generates a random backoff in the window [0 − *W*_0_] before sensing the carrier. The busy channel probability for the following fragment, block, or CoAP ACK is given by the probability that no other node accesses the channel during (*W*_0_ + 1)/2 time units. In average terms, the channel will be busy if no other nodes accessed and found it idle in the previous *L_eq_* time units:

(4) $$L_{eq}=(L+F\times (W_{0}+1)/2)/(F+1)\;\text{for}\;6\text{LoWPAN Fragmentation}$$

(5) $$L_{eq}=\left(L+\left(2\;B-1\right)\times \left(W_{0}+1\right)/2\right)/(2\;B)\;\text{for Blockwise transfer}\;$$

We recall that the MAC ACK is transmitted right after the reception of a MAC frame. Its transmission does not undergo the backoff procedure. Instead, this procedure applies for the CoAP ACK. For 6LoWPAN Fragmentation there are F packets and 1 potential CoAP ACK, while for CoAP blockwise transfer there are B blocks and B potential CoAP ACKs.

The busy channel probability at the first CCA can be evaluated as:

(6) $$\alpha_{l,0}=\;L_{eq}\sum_{i=1}^{N-1}\sum_{q=1}^{C_{l,i}}\prod_{k=1}^{i}\tau_{k_{q}}\prod_{h=1+1}^{N-1}\left(1-\tau_{h_{q}}\right)\;\left(1-\prod_{k=1}^{i}{\bar{\alpha }}_{k_{q}}\right)\;$$

The double sum in *i* and *q* enumerates the combinations of events in which *i* nodes access the channel in a given time unit (excluding the transmitting node l). The term $\left(1-\prod_{\text{k}=1}^{\text{i}}{\bar{\alpha }}_{{\text{k}}_{\text{q}}}\right)$ considers the events of successful CCAs.

Given *N* nodes in the network, the subscript *k_q_* refers to the node in the *k*-th position in the *q*-th combination of *i* out of *N-1* elements.

Should the channel be busy during the first backoff, there is a higher probability that the channel will be still busy after the backoff in the window [0 − *W*_1_]. This behavior is due to the bursty traffic generation. Under this condition, the channel will be busy at the second CCA with a probability:

(7) $$\alpha_{l,1}=1-((W_{1}+1)/2)/(\bar{L}+(W_{1}+1)/2)$$

where:

(8) $$\begin{array}{c} \bar{L}=(F\times L+L_{\text{ACK}})/(F+1)\;\text{for}\;6\text{LoWPAN fragmentation}\\ \bar{L}=(L+L_{\text{ACK}})/2\;\text{for blockwise transfer} \end{array}$$

This condition holds for all the backoff attempts in which the backoff window is lower than the size of the burst. Therefore, Equation (7) can be generalized for a generic backoff stage *j* such that for *W_j_* < *L* × *F* or *W_j_* < *L* × *B*.

For *W_j_* > *L* × *F* or *W_j_* > *L* × *B* the busy channel probability α*_l,j_* can be calculated in asynchronous fashion, as in the original model in [14].

In conclusion, the busy channel probability is written as for *j* > 0

(9) ={Leq∑i=1N−1∑q=1Cl,i∏k=1iτkq(1−∏k=1iα¯kq)∏h=1+1N−1(1−τhq)forWj>Fλ,Bλ1−(Wj+1)2(L¯+(Wj+1)2)−1otherwiseαl,j

where $C_{l,i}=\left(\begin{array}{c} l\\ i \end{array}\right)$ and ${\bar{\alpha }}_{l}=\frac{\sum_{j=0}^{m}\alpha_{l,j}}{m+1}$ is the average busy channel probability for all backoff stages.

Notice that this expression is an approximation for the busy channel probability when W_j_ ≈ L×F or W_j_ ≈ L×B.

The collision probability is the probability that a contending node performs the CCA in the same time unit, *i.e.*:

(10) $$P_{\text{coll},l}={\bar{\alpha }}_{l}/L_{eq}$$

The expressions of the CCA probability, the busy channel probability and the collision probability form a system of non-linear equations that can be solved through numerical methods as specified in [14].

The IEEE 802.15.4 protocol does not distinguish between higher layer packets. Therefore, the probability that the transmission of a block, fragment or CoAP ACK fails has the same expression for each of them. We refer to it as P_frame,l_:

(11) $$P_{\text{frame},l}=P_{cf,l}+P_{cr,l}$$

where P_cf,l_ corresponds to the probability for node *l* that the frame is discarded due to channel access failure and P_cr,l_ to the probability for node *l* of a packet to be discarded due to retry limit. Therefore we have

(12) $$P_{cf,l}=\prod_{j=0}^{m}\alpha_{l,j}\sum_{k=0}^{n}P_{\text{coll},l}\left(1-\prod_{j=0}^{m}\alpha_{l,j}\right)$$

Once the CCA probability, the busy channel probability, and the collision probability are derived, the delay for successfully received frames D*_frame,l_* and CoAP ACKs D*_ACK,l_* are obtained as follows.

(13) $${\;\text{D}}_{\text{frame},l}=\frac{\sum_{n}^{h=0}P_{\text{coll},l}^{h}{\left(1-\prod_{m}^{j=0}\alpha_{l,j}\right)}^{h}\left(\sum_{h}^{x=0}E\left\lceil T_{x}\right\rceil +\left(h+1\right)T_{\text{frame}}\right)}{\sum_{k=0}^{nr}\left(P_{\text{coll},l}\left(1-\prod_{m}^{j=0}\alpha_{l,j}\right)\right)k}$$

where *T_x_* is the backoff stage delay, whereas *T_frame_* is the time periods in number of time units for MAC frame and MAC ACK transmission.

The denominator of Equation (13) is a normalization factor with respect to the probability of successful reception. Since the backoff time in each stage k is uniformly distributed in [0, W_k_ − 1], the expected total backoff delay is:

(14) $$E\left\lceil T_{l}\right\rceil =1+\frac{\sum_{r=0}^{m}{\bar{\alpha }}_{l_{l}}^{r}\left(1-{\bar{\alpha }}_{l}\right)\left(r+\sum_{k=0}^{r}\frac{W_{k}-1}{2}\right)}{\left(1-\prod_{j=0}^{m}\alpha_{l,j}\right)}$$

The expression of D_ACK,l_ is obtained by replacing T_frame_ with TACK, being the time periods in number of time units for CoAP ACK and MAC ACK transmission. The denominator of Equation (14) is a normalization factor with respect to the probability of idle channel within the maximum number of backoff.

## 4. Analytical Model

In this section we present the analytical model of CoAP data transactions that use 6LoWPAN fragmentation and CoAP blockwise transfer. We also derive the expression of the transition probabilities that characterize the model. The reference WSN topology is a star network. In this scenario, a client is in direct communication (single-hop) with the servers that are in the network. The analytical model is present only in the Client and Server nodes. As explained next, we model these nodes with two separate Markov chains.

**Figure 5.**
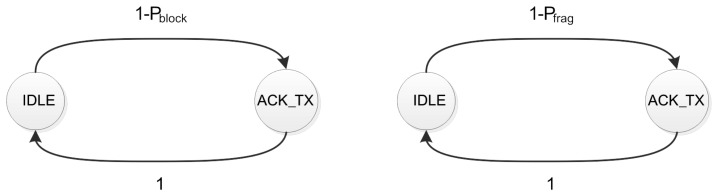
Blockwise transfer in observe data transaction. Two blocks compose each update. In the second update the CoAP ACK sent by the client fails while in the third one the second block fails. Both failures cause the retransmission of the relative block.

The analytical model of the client is shown in Figure 5. It is composed by two states, which are the same for the CoAP blockwise transfer and 6LoWPAN fragmentation. The only difference is the transition probabilities between the states.

The first state is the IDLE, which means that the client is waiting for the reception of a block or for the F fragments of an update. The client visits the acknowledgment transmission state (CoAP ACK_TX) after it receives successfully a block or all the F fragments. In both cases it sends the relative CoAP ACK.

The transition probabilities of each chain are equivalent to the probability of receiving correctly a block or all the fragments of an update.

The probability P_block,l_ that a single block fails at node *l* is therefore equal to:

(15) $$P_{\text{block},l}=P_{\text{frame},l}$$

where *P_frame,l_* is derived in Equation (11).

Instead, the probability P_frag,l_ that the transmission of a fragmented update fails is equal to:

(16) $$P_{\text{frag},l}=1-{\left(1-P_{\text{frame},l}\right)}^{F}$$

**Figure 6.**
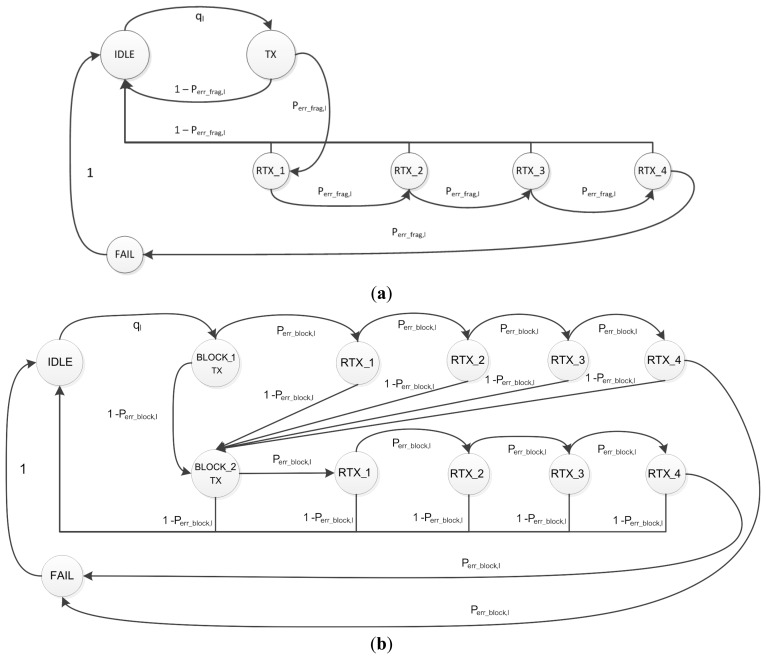
Markov chains for the server. (**a**) 6LoWPAN Fragmentation case (**b**) Blockwise transfer case. (**a**) The server retransmits all the fragments if the transmission of any of them fails or it does not receive the CoAP ACK; (**b**) The server retransmits a single block if its transmission fails or it does not receive the relative CoAP ACK. The Markov chain represents the transmission of an observe update composed by two blocks.

Figure 6 shows the server's Markov chains for CoAP blockwise transfer and 6LoWPAN fragmentation. Figure 6b considers the transmission of an observe update composed by two blocks. Both chains have four retransmission states, which correspond to the maximum number of retransmissions defined by CoAP. The blockwise transfer model has a transmission and a retransmission stage for each block that composes the update. The CoAP layer, in fact, controls the transmission of each block and sends the subsequent only after the previous one has been acknowledged by the client. The CoAP layer, instead, has a single transmission and retransmission stage when the update is transmitted with 6LoWPAN fragmentation.

The server is in the IDLE state when it is waiting for the generation of the next update. The transition probability from the IDLE to the transmission state of the fragmented update (TX) or the first block (BLOCK_1 TX) is the probability *q_l_* that we derived in Equation (1). The server goes back to IDLE state after it receives the CoAP ACK relative to the last block or fragmented update. The failure of the update transmission also causes the server to go back to the IDLE state.

The retransmission states are visited after the failure of the transmission of a block, fragment or CoAP ACK. The transition probability between the transmission state and the first retransmission state of the blockwise and fragmentation models are expressed as $P_{{\text{err}}_{\text{block}},l}$ and $P_{{\text{err}}_{\text{frag}},l}$, respectively. These probabilities are also valid for the transition between the retransmission states.

The probabilities at node *l* $P_{{\text{err}}_{\text{block}},l}$ and $P_{{\text{err}}_{\text{frag}},l}$ that a block or a fragmented update is retransmitted are defined as follows:

(17) $$P_{{\text{err}}_{\text{block}},l}=P_{\text{block},l}+P_{{\text{ack}}_{\text{block}},l}$$

(18) $$P_{{\text{err}}_{\text{frag}},l}=P_{\text{frag},l}+P_{{\text{ack}}_{\text{frag}},l}$$

where $P_{{\text{ack}}_{\text{block}},l}$ and $P_{{\text{ack}}_{\text{frag}},l}$ are the probabilities at node *l* that the transmission of the CoAP ACK relative to a block or to the fragmented update fails, respectively. These are equal to:

(19) $$P_{{\text{ack}}_{\text{block}},l}=P_{\text{frame},l}\times (1-P_{\text{block},l})$$

(20) $$P_{{\text{ack}}_{\text{frag}},l}=P_{\text{frame},l}\times \;(1-P_{\text{frag},l})$$

The unsuccessful retransmission of a block or of the fragmented update for *c* consecutive times causes the update transmission to fail and the server to visit the FAIL state.

The probabilities $P_{{\text{fail}}_{\text{block}},l}$ and $P_{{\text{fail}}_{\text{frag}},l}$ that an update transmission fails is equal to the probability that the transmission as well as the retransmissions of the update fail. These are equal to:

(21) $$P_{{\text{fail}}_{\text{block}},l}=\sum_{i=1}^{B}{P_{{\text{err}}_{\text{block}},l}}^{c+1}\times {\left(1-{P_{{\text{err}}_{\text{block}},l}}^{c+1}\right)}^{i-1}$$

(22) $$P_{{\text{fail}}_{\text{frag}},l}={P_{{\text{err}}_{\text{frag}},l}}^{c+1}$$

In the rest of this section we present the expressions for the performance metrics that we use to evaluate CoAP blockwise transfer and 6LoWPAN fragmentation.

### 4.1. Reliability

WSN applications that monitor a critical environment or a critical physical variable require that the data collected by sensor nodes must be delivered reliably to destination. However, wireless links are error prone and ensuring end-to-end reliable data transfer is one of the major challenges in WSNs. In the proposed model we define reliability as the probability that the update sent by a CoAP server arrives correctly at destination.

In the previous section we derived the probability that the transmission of an update fails. Next we derive the expression of the end-to-end reliability for the CoAP blockwise transfer R_block,l_ and the 6LoWPAN fragmentation R_frag,l_.

(23) $$R_{\text{block},l}=1-\;P_{{\text{fail}}_{\text{block}},l}$$

(24) $$R_{\text{frag},l}=1-\;P_{{\text{fail}}_{\text{frag}},l}$$

### 4.2. Latency

The latency that can be tolerated by an application is of paramount importance to choose the appropriate data transfer technique. WSN applications could have strict deadline requirements on the data sensed by a device. Scenarios such as e-Health or industrial monitoring are an example of those applications. In this paper, we define latency as the time required to complete a data transaction between server and client. The reception of the CoAP ACK relative to the fragmented update or the last block determines the end of the transaction.

The latency of a CoAP transaction has to consider the delay caused by an unsuccessful frame transmission. The value of the RTO includes this delay. The expression of the delay of a frame transmission was derived in the previous section. Next we present the latency for each case:

#### 4.2.1. Latency for 6LoWPAN fragmentation

The latency of an update transmission that uses 6LoWPAN fragmentation is equal to the sum of the transmission delay of the CoAP ACK and that of each fragment. We define the latency for 6LoWPAN fragmentation D_frag,l_ as:

(25) $$D_{\text{frag},l}=\sum_{j=0}^{c}\text{Pr}\left({ℱ}_{j}|ℱ\right)\;D_{j}$$

where Pr( 
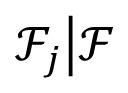
) is the probability of successful update transmission at the *(j + 1)th* attempt given a successful update transmission within *(c + 1)* attempts.

(26) $$\mathrm{Pr}\left({ℱ}_{j}|ℱ\right)=\left(1-P_{{\text{err}}_{\text{frag}}}\right)P_{{\text{err}}_{\text{frag}}}{}^{j}/R_{\text{frag}}$$

*D_j_* is the delay of an update that is successfully transmitted at the *(j + 1)th* attempt, *i.e.*,

(27) $$D_{j}=D_{\text{ACK},l}+f*D_{\text{frame},l}+j\times (\text{RTO}+D_{\text{ACK},l}+fD_{\text{frame},l})$$

where D_CoAP ACK,l_ is the delay of the CoAP ACK transmission, D_frame,l_ is the delay of a fragment at node *l* and RTO is the value of the CoAP retransmission timeout.

#### 4.2.2. Latency for Blockwise transfer

The latency of an update transmission using the CoAP blockwise transfer is defined as follows:

(28) $$D_{\text{block},l}=B\sum_{j=0}^{c}\text{Pr}(L_{j}|L)\;D_{j}$$

where Pr(ℒ_j_|ℒ) is the probability of successful block transmission at the *(j* + *1)th* attempt given a successful block transmission within *(c* + *1)* attempts.

(29) $$\mathrm{Pr}\left({ℒ}_{j}|ℒ\right)=\left(1-P_{{\text{err}}_{\text{block}}}\right)P_{{\text{err}}_{\text{block}}}{}^{j}/R_{\text{block}}$$

*D_j_* is the delay of a block that is successfully transmitted at the *(j* + *1)th* attempt, *i.e.*,

(30) $$D_{j}=D_{\text{ACK},l}+D_{\text{frame},l}+j\times \left(\text{RTO}+D_{\text{ACK},l}+D_{\text{frame},l}\right)$$

## 5. Performance Evaluation

In this section we validate the model by Monte Carlo simulations and present the results of the performance evaluation. We base the simulation parameters on the specification of the IEEE 802.15.4 [2] and CoAP [7] protocols. We evaluate our models with different values of the traffic pattern and for CoAP updates composed by a variable number of fragments and blocks. The MAC parameters are selected as *m_0_* = *3*, *m_B_* = *5*, *m* = *4*, *n* = *0*, in accordance with the 802.15.4 standard [2]. The MAC frames have size *L* = *L_ACK_* = *127 bytes* and the MAC ACK frame *L_MAC ACK_* = *11 byt*es. We study various traffic and fragmentation scenarios by considering *N* = *[10, 15, 20]* nodes with update generation rates *λ* = *[0.1… 1]* pkt/s ad updates divided into *B* = *[1, 3, 5, 7]* blocks (in CoAP blockwise transfer) or *F* = *[1, 3, 5, 7]* fragments (in 6LoWPAN fragmentation). As previously mentioned, the generation rate *λ* is constrained by the value of the minimum RTO recommended by CoAP [7]. In this sense, the timeout is *RTO* = *1s* with a random backoff of 0.5s. The number of retransmission is set as *c* = *1*.

### 5.1. Reliability

**Figure 7.**
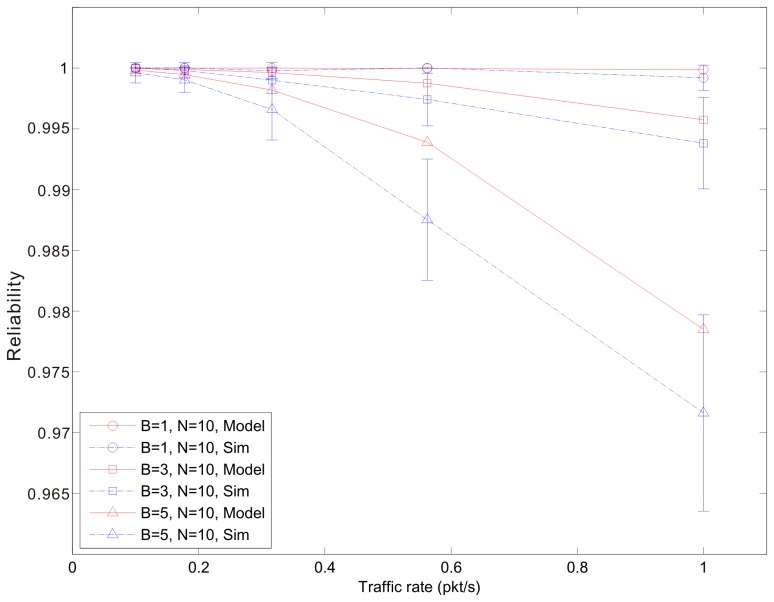
Blockwise reliability *versus* traffic rate for a star topology network composed by 10 nodes.

**Figure 8.**
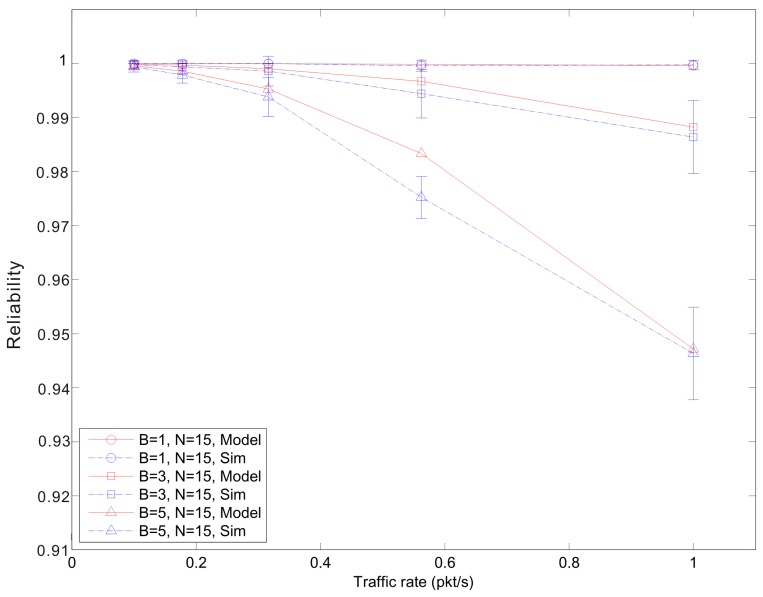
Blockwise reliability *versus* traffic rate for a star topology network composed by 15 nodes.

**Figure 9.**
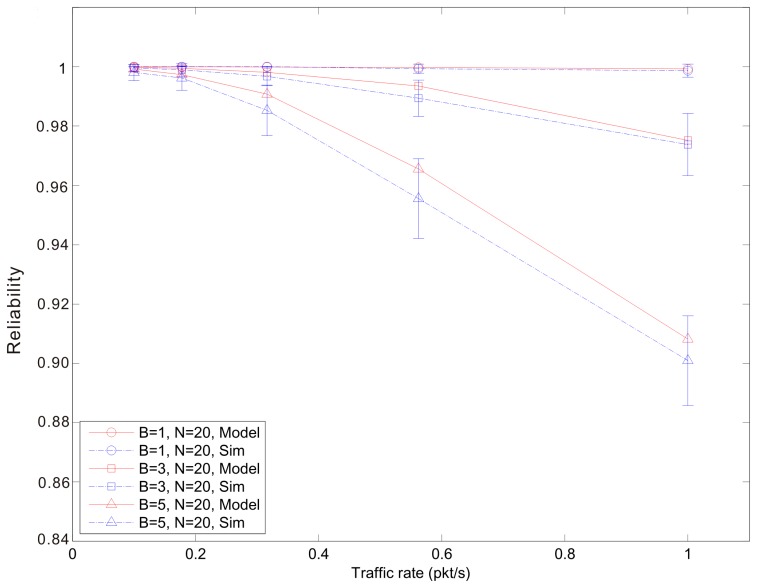
Blockwise reliability *versus* traffic rate for a star topology network composed by 20 nodes.

**Figure 10.**
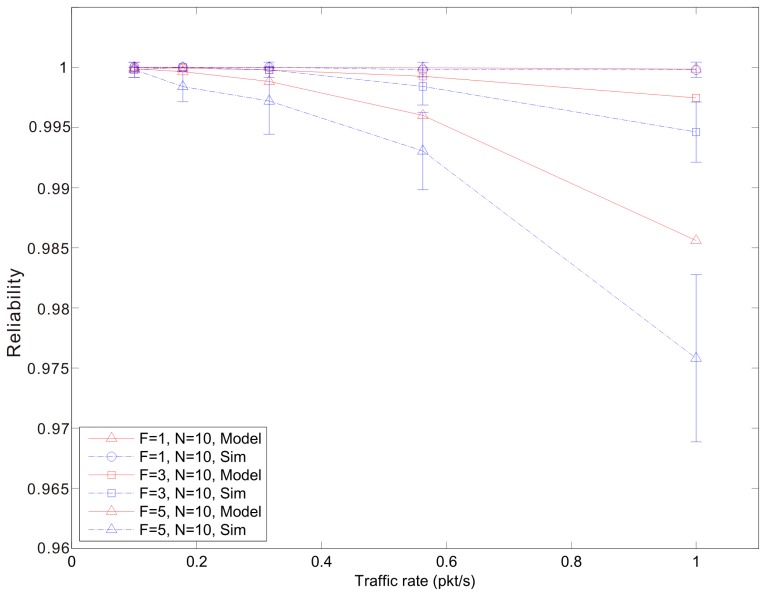
6LoWPAN fragmentation reliability *versus* traffic rate for a star topology network composed by 10 nodes.

**Figure 11.**
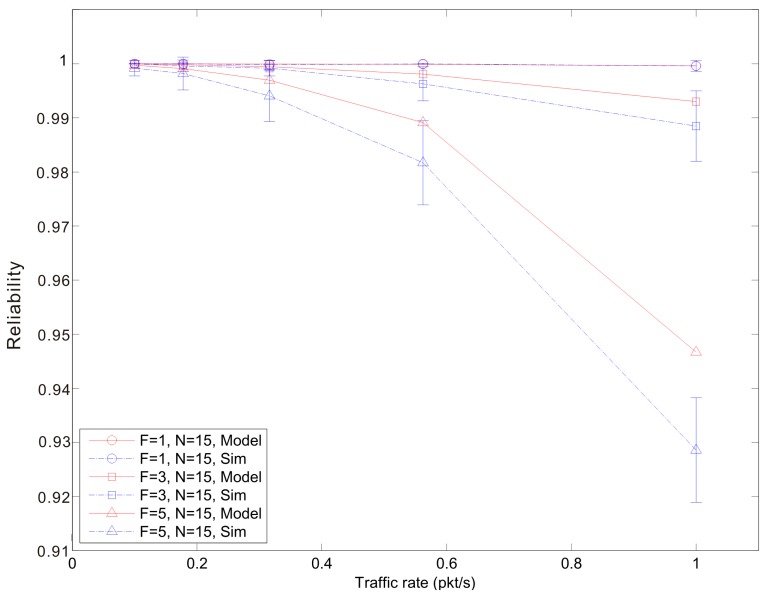
6LoWPAN fragmentation reliability *versus* traffic rate for a star topology network composed by 15 nodes.

**Figure 12.**
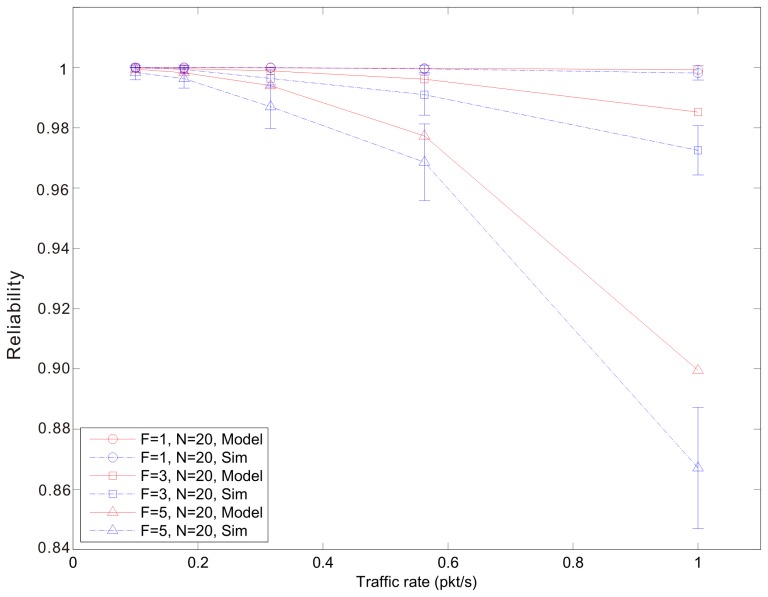
6LoWPAN fragmentation reliability *versus* traffic rate for a star topology network composed by 20 nodes.

Figures 7, 8 and 9 show the average reliability of CoAP blockwise transfer computed over all the links for a star topology network with mixed traffic conditions. Figures 10, 11 and 12 show the average reliability of 6LoWPAN fragmentation for the same scenario. A good agreement between simulations and analytical results of the model is observed. The reliability performance of 6LoWPAN fragmentation and CoAP blockwise transfer is very similar in the considered scenarios. The difference between the reliability values is lower than 2%. In particular, CoAP blockwise transfer has a slightly better reliability when the traffic conditions congest the WSN, which is the case for *N* = 20, *B* = 5 and λ greater than 0.8 pkt/s. In these conditions blockwise improves reliability by the 0.96% respect to 6LoWPAN fragmentation.

However, 6LoWPAN fragmentation is slightly more reliable than blockwise when the traffic conditions do not congest the WSN. However, for *N* = 10 the trends of both solutions are very close and differ by the 0.2% for λ = 1 pkt/s and *F* = 3. This difference grows up to the 0.7% for *F* = 5 with the same traffic rate. For *N* = 15 6LoWPAN fragmentation has a maximum improvement of the 1% over blockwise, which is obtained for λ = 1 pkt/s and *F* = 5. A similar difference is observed for *N* = 20, *F* = 3 and the same traffic rate.

**Figure 13.**
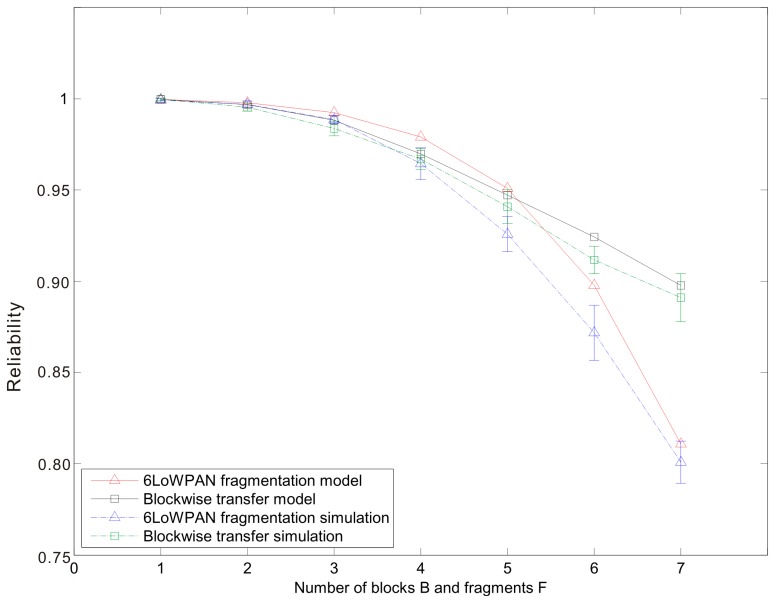
Blockwise and 6LoWPAN reliability *versus* the number of blocks or fragments that compose an update. The network is composed by 15 nodes and the traffic rate is fixed to 1 pkt/s.

The same behavior can be observed in Figure 13, which shows the average reliability of CoAP blockwise transfer and 6LoWPAN fragmentation according to the variation of the number of blocks or fragments that compose an update. The average reliability is computed over all the links for a star topology network of *N* = 15 nodes and λ = 1 pkt/s. 6LoWPAN fragmentation improves slightly reliability for a number of fragments lower than five. The reliability trend of 6LoWPAN fragmentation undergoes a pronounced drop when the number of fragments grows and the WSN becomes more congested. In this situation, CoAP blockwise transfer has a less pronounced drop, which allows outperforming 6LoWPAN fragmentation. In particular, for *B* = 7 blockwise transfer improves reliability by the 10.7% respect to fragmentation. In congested WSNs, in fact, the probability that a fragment or block is retransmitted is high. Therefore, consecutive failures of blocks belonging to the same update do not cause the failure of the update transmission, as it would happen in 6LoWPAN. CoAP Blockwise transfer, therefore, is able to reduce the number of lost updates establishing a reliable transfer for each single block.

In CoAP blockwise transfer, the transmission of a CoAP ACK for each block causes an increase of the channel occupancy. This has a counter-effect on the reliability that is more evident when the network is not congested. In this situation, CoAP blockwise transfer increases the average network traffic augmenting the collision probability. 6LoWPAN fragmentation requires the transmission of fewer messages for a single update. It is able, therefore, to reduce the network traffic and the retransmission probability of an update. A node using 6LoWPAN fragmentation is able to reduce significantly the occupancy of the channel. Thereby, the probability that a concurrent node finds the channel busy when attempting the transmission is lower respect to CoAP blockwise transfer. Besides the higher probability of finding the channel idle, a fragment has less chance to collide with the transmission of another one or with a CoAP ACK. 6LoWPAN fragmentation is able, therefore, to improve reliability under these traffic conditions.

### 5.2. Latency

**Figure 14.**
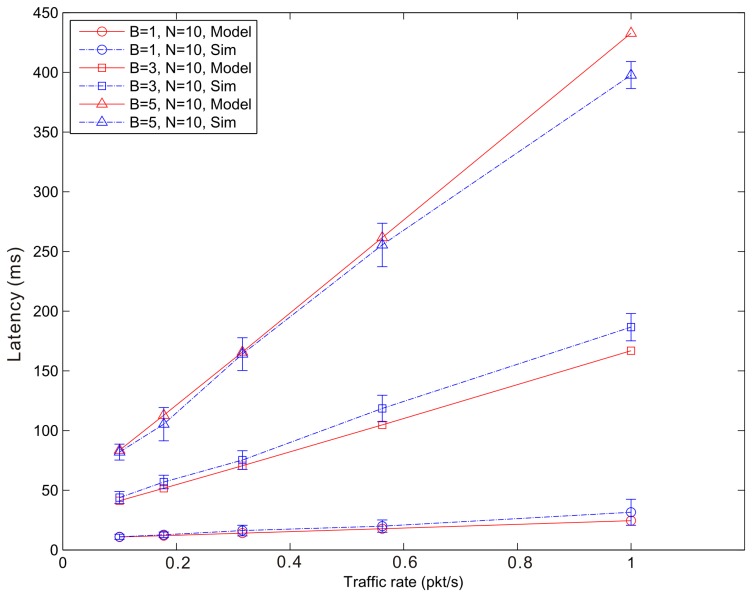
Blockwise Latency *versus* traffic rate for a star topology network composed by 10 nodes.

**Figure 15.**
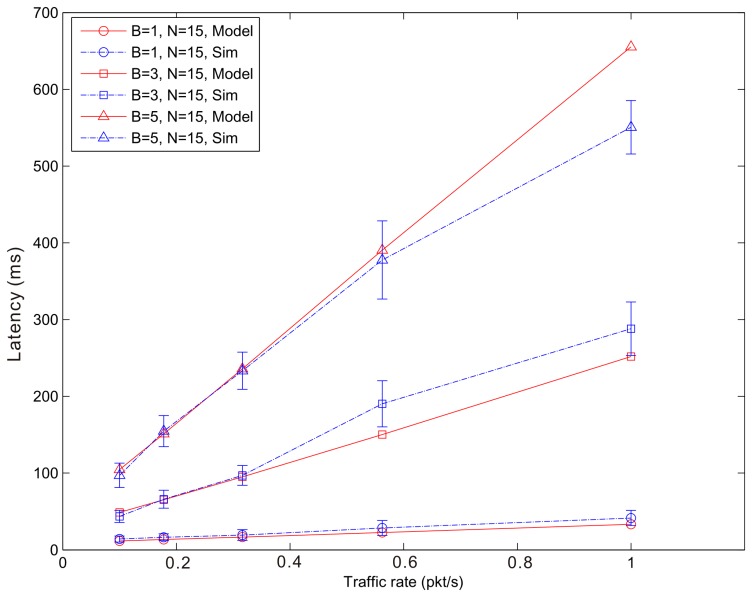
Blockwise Latency *versus* traffic rate for a star topology network composed by 15 nodes.

**Figure 16.**
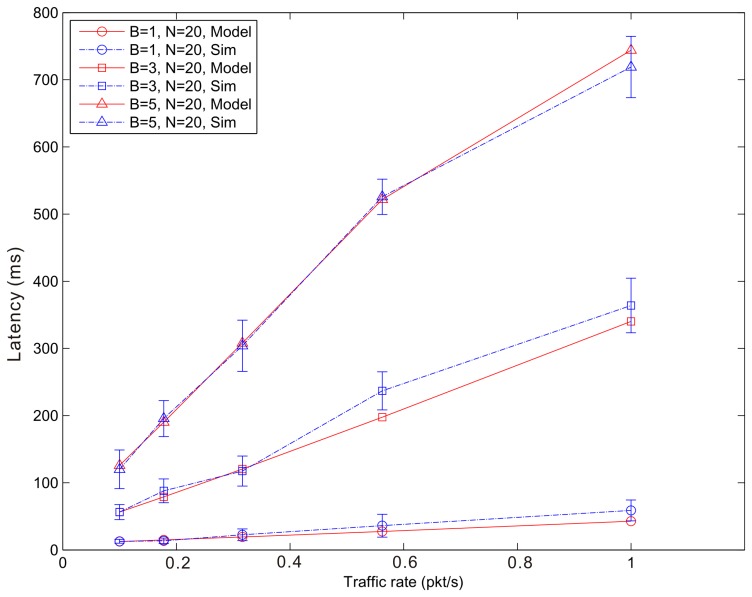
Blockwise Latency *versus* traffic rate for a star topology network composed by 20 nodes.

**Figure 17.**
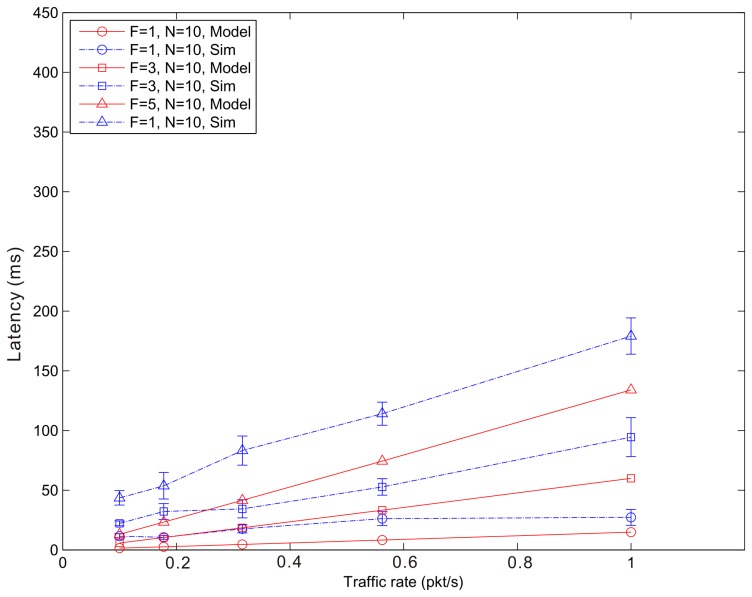
6LoWPAN Fragmentation Latency *versus* traffic rate for a star topology network composed by 10 nodes.

**Figure 18.**
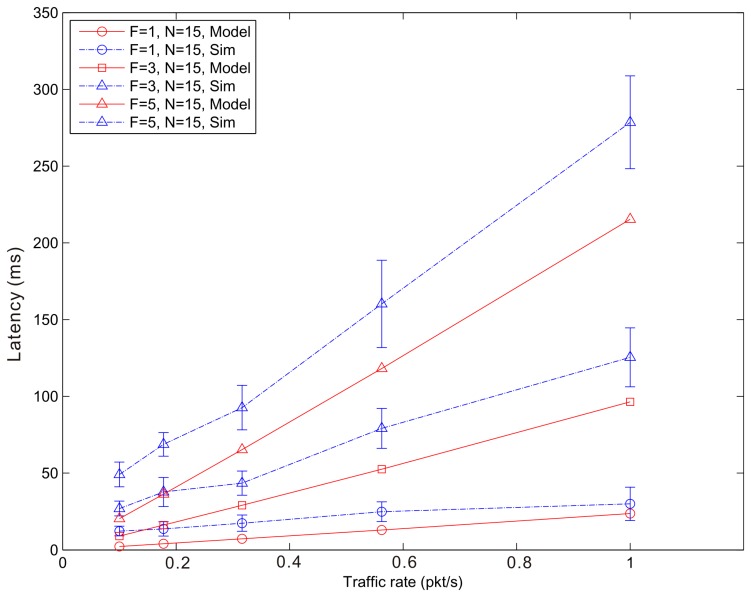
6LoWPAN Fragmentation Latency *versus* traffic rate for a star topology network composed by 15 nodes.

**Figure 19.**
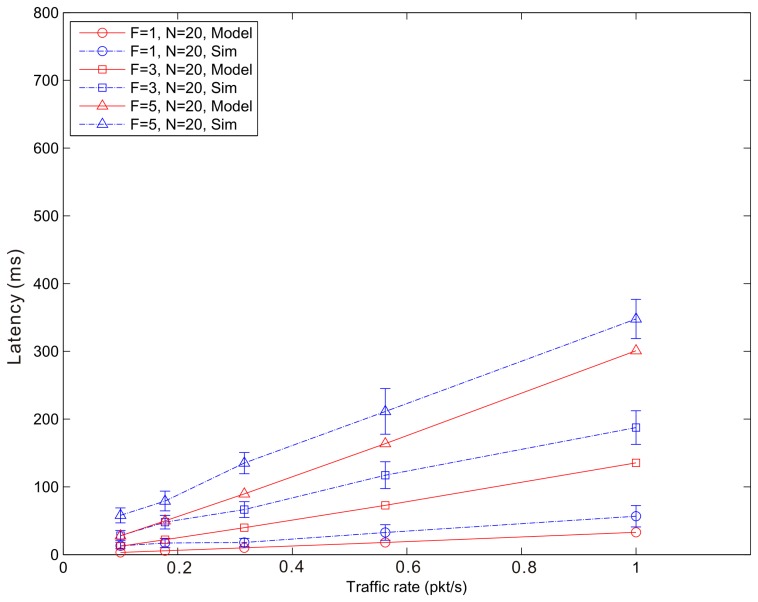
6LoWPAN Fragmentation Latency *versus* traffic rate for a star topology network composed by 20 nodes.

Figures 14, 15 and 16 show the average latency of CoAP blockwise transfer computed over all the links for a star topology network with mixed traffic conditions. Figures 17, 18 and 19 show the average latency for 6LoWPAN fragmentation. A good agreement between simulations and analytical results of the model is observed.

According to our performance evaluation, 6LoWPAN fragmentation outperforms CoAP blockwise transfer in terms of latency independently from the update generation rate and the number of nodes. The difference between both techniques becomes higher with the growth of the update generation rate and the number of fragments or blocks involved in the communication. As mentioned, 6loWPAN fragmentation requires the interchange of fewer messages than CoAP blockwise transfer. Consequently, the latency of an update transmission is significantly lower than that experienced by CoAP blockwise transfer. The performance of CoAP blockwise transfer, however, could be improved by considering block sizes that allow sending a single block in more than one frame. This would reduce the number of CoAP ACKs and consequently the latency performance would improve. However, its performance would be always lower than that of 6LoWPAN fragmentation, which represents an upper bound to the performance of CoAP blockwise transfer. 6LoWPAN fragmentation could be considered as a particular case of CoAP blockwise transfer with a block size that allows sending a single CoAP ACK.

**Figure 20.**
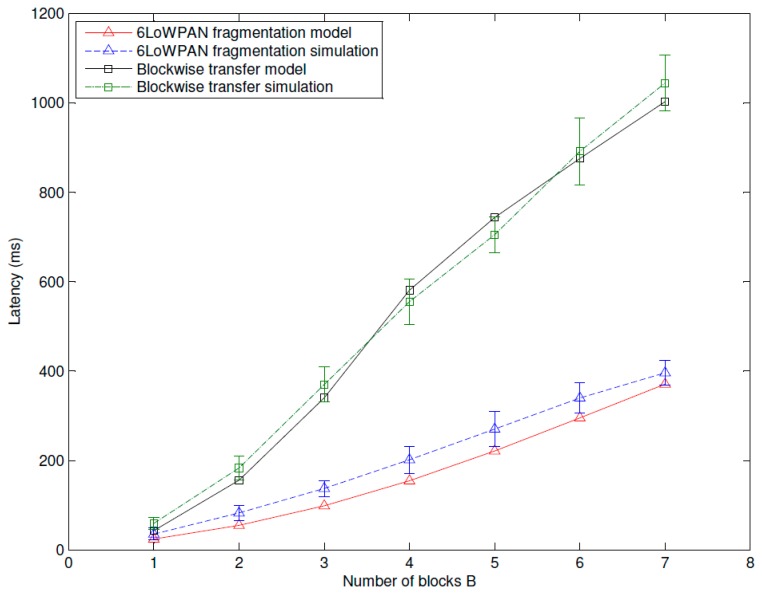
Blockwise and 6LoWPAN latency *vs.* the number of blocks or fragments for a star topology network composed by 20 nodes and a traffic rate of 1 pkt/s.

The latency of CoAP blockwise transfer has a sharp rise for increasing values of the traffic rate, which further augments the difference with that of 6LoWPAN fragmentation. The same behavior can be observed in Figure 20, which shows the latency trend according to the number of blocks or fragments of an update in a WSN composed by 15 nodes and traffic rate of 1 pkt/s. The growth of the traffic rate as well as that of the number of fragments (6LoWPAN fragmentation) or blocks (CoAP blockwise transfer) congest the WSN and augment the retransmission probability of an update. Although in case of congestion CoAP blockwise transfer shows a slightly better reliability, the cost in terms of latency of block-to-block retransmission does not allow improving its performance. The retransmission of the entire update in 6LoWPAN fragmentation has less effect on the average latency.

**Figure 21.**
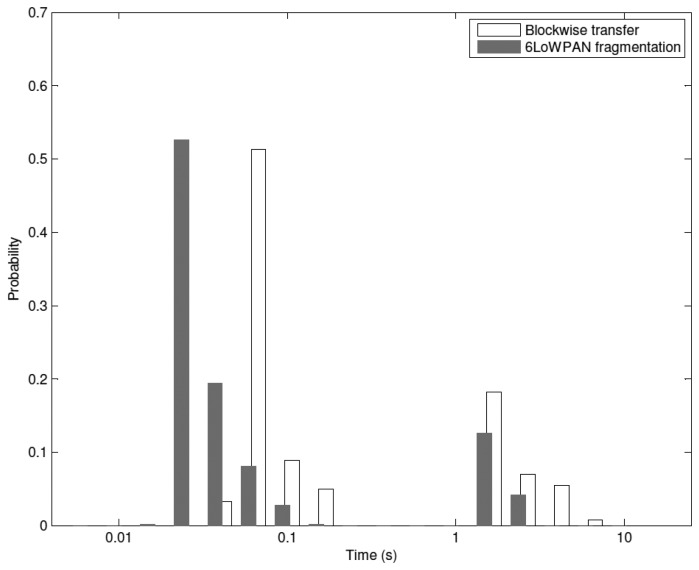
PDF of the latency for a star topology network with 15 nodes and a traffic rate of 1 pkt/s. 6LoWPAN. Each update is composed by five fragments or blocks. For the sake of clarity, the *x*-axis is shown in logarithmic scale.

This behavior can be explained analyzing the Probability Density Function (PDF) of the latency, which is shown in Figure 21. It is evaluated in a star topology WSN composed by 15 nodes with a traffic rate of 1 pkt/s and updates composed by five fragments or blocks. The distribution of both solutions presents a long tail, which is due to the effect of retransmissions. The presence of block-to-block retransmissions causes the tail of CoAP blockwise transfer to be the longest one. This further worsens its average latency and causes the rapid growth of its curve. In CoAP blockwise transfer each block of an update could be retransmitted. The overall latency would be higher than that of retransmitting the entire update as done by 6LoWPAN fragmentation. We remark that reliability and latency can be competing requirements and a trade-off is needed to guarantee low energy consumption. The model can be used within a constrained optimization framework for the selection of protocol parameters and fragmentation techniques.

### 5.3. Model Limitations

Here, we discuss the fundamental limitations of the analytical model developed and analyzed in the previous sections. First, we remark that the Markov chain model requires the solution of systems of non-linear equations to derive MAC indicators such as the CCA probability, the busy channel probability and the collision probability in Section 3.3. For the use of such a model for online computation in real sensors, the complexity is a critical factor since the typical micro-controller does not support well a complex computing. In heterogeneous network conditions, a Markov chain has to be solved for each link, and the complexity increases with the number of links. The use of approximated model equations is advocated in [14], when the number of nodes exceeds 15, to guarantee bounded computation times in the order of seconds for typical sensor platforms.

The model includes the effects of bursty traffic on the busy channel probability in different backoff stages. However, we assume an average CCA probability *τ_l_* in each time unit. As we see from the simulation results, this is a fair approximation when the number of blocks (or fragments) is limited. When the traffic in the network becomes saturated, the performance of the MAC layer is influenced also by higher order statistics of *τ_l_*.

A practical limitation with high traffic conditions is also given by the retransmission mechanism of CoAP that defines a minimum RTO in the order of 1 s. This is specified by the standard to guarantee support for multi-hop communications. However, that limits the derivation of the offered traffic in the network, since the packet service time increases quickly with the update generation time, especially for block-wise transfer, where the retransmission is performed on a block-level, as we saw in Figure 20.

## 6. Conclusions

In this paper, we have analyzed CoAP data transactions with large payloads in WSN with star topology. We have proposed a novel analytical model to study the performance of 6LoWPAN fragmentation and CoAP blockwise transfer. We have adopted reliability and latency as performance indicators. We have used Monte Carlo simulation to validate our model. The results demonstrate accuracy to estimate the performance of CoAP blockwise transfer and 6LoWPAN fragmentations.

As for reliability, we have observed a good performance of both techniques with small differences between them. However, depending on the traffic conditions a technique could be preferred to the other. In particular, CoAP blockwise transfer is a more reliable solution when traffic conditions lead to a congestion of the WSN, which is the case of applications with high traffic rates or that produce updates composed by many blocks. 6LoWPAN fragmentation is preferable when the WSN links are less congested.

A clear disadvantage of CoAP blockwise transfer is the latency introduced by acknowledging each single block, which worsen the performance of CoAP blockwise transfer. According to our results 6LoWPAN fragmentation outperforms CoAP blockwise transfer in terms of latency also in congested WSN.

In conclusion, applications that have strict requirements in terms of latency, *i.e.*, real-time applications, should adopt 6LoWPAN fragmentation. With more relaxed constraints on latency, *i.e.*, applications that use data logging, CoAP blockwise transfer should be adopted when the traffic conditions are close to saturation. A good trade-off could be reached using an algorithm able to choose dynamically which technique use depending from the traffic conditions.

## Figures and Tables

**Table 1.** Main symbols used in this paper.

**Symbols**	**Meaning**
λ*_l_*	Traffic generation probability at CoAP layer
α*_l,j_*	Busy channel probability of node l at the backoff stage j
${\bar{\alpha }}_{l}$	Average busy channel probability for all backoff stages
*τ_l_*	Channel access probability of node l
${\text{b}}_{0,0,0}^{\left(\text{l}\right)}$	Idle probability of node l
c	Maximum number of CoAP retransmissions
m_0_	Initial backoff exponent macMinBE
m_B_	Maximum backoff exponent macMaxBE
m	Maximum number of backoff macMaxCSMABackoffs
n	Maximum number of MAC retransmissions macMaxFrameRetries
*q_l_*	Probability of generating the first fragment or block of an update at node l
*S_b_*	Backoff unit time aUnitBackoffPeriod
N	Number of nodes in the network
F	Number of 6LoWPAN fragments composing an update
B	Number of CoAP blocks composing an update
L	Fragment or block size
L_ACK_	CoAP ACK size
L_MAC ACK_	MAC ACK size
*L_eq_*	Vulnerability window for the CCA
*P_coll,l_*	Collision probability of node l
P_frame,l_	Probability that the transmission of a block, fragment or CoAP ACK for node l fails
P_cf,l_	Probability for node l that the frame is discarded due to channel access failure
P_cr,l_	Probability for node l of a frame to be discarded due to MAC retry limit
P_block,l_	Probability that a single block fails at node l
P_frag,l_	Probability that a 6LoWPAN fragmented update fails at node l
P_errblock,l_	Probability that a block is retransmitted at node l
P_errfrag,l_	Probability that a 6LoWPAN fragmented update is retransmitted at node l
P_ackblock,l_	Probability that the transmission of the CoAP ACK relative to a block transmission fails
P_ackfrag,l_	Probability that the transmission of the CoAP ACK relative to a 6LoWPAN fragmented update fails
*P_failblock,l_*	Probability that an update sent using blockwise transfer fails
*P_failfrag,l_*	Probability that an update sent using 6LoWPAN fragmentation fails
*R_block,l_*	End-to-end reliability for blockwise transfer
*R_frag,l_*	End-to-end reliability for 6LoWPAN fragmentation
*D_frag,l_*	Delay of a successful received 6LoWPAN fragmented update
*D_block,l_*	Delay of a successful received update using blockwise transfer
*D_frame,l_*	Delay for successfully received frame
*D_ACK,l_*	Delay for successfully received CoAP ACK
RTO	CoAP retransmission timeout
